# High Non-COVID-19 in-Hospital Deaths during the First Lockdown in Israel Compared with the Second and Third Lockdowns

**DOI:** 10.3390/ijerph192013134

**Published:** 2022-10-12

**Authors:** Shiran Bord, Aviad Tur-Sinai, Fuad Basis

**Affiliations:** 1 Department of Health Systems Management, The Max Stern Yezreel Valley College, Yezreel Valley 1930600, Israel; 2 School of Nursing, University of Rochester Medical Center, Rochester, NY 14642-8404, USA; 3 Rambam Health Care Campus, Haifa 3109601, Israel; 4 Faculty of Medicine, Technion Israel Institute of Technology, Haifa 3200003, Israel

**Keywords:** admission, discharges from hospital, hospital deaths, lockdown, COVID-19

## Abstract

During the first lockdown in Israel, citizens were instructed to visit community clinics only for urgent cases. However, they were not informed that emergency departments (EDs) were safe. Reports from the National Ambulance Services showed a 22% increase in at-home deaths during the lockdown. Perhaps, the reason is because some critically ill patients postponed referrals and came “at the last minute”. After the first lockdown, the Ministry of Health (MOH) declared that hospital EDs were safe. The objective of the study was to examine the rates of admission from EDs to hospital wards, and non-COVID-19 in-hospital deaths during the first lockdown in Israel, compared with the second and third lockdowns. From the business intelligence software of the Rambam Medical Center in Israel, we collected data about the rates of admission to the ED, the non-COVID-19 in-hospital deaths during the three lockdowns, during the same periods in the previous three years, and the main five causes of non-COVID-19 deaths. Data comparison was done using multiple chi-square tests. ED admission numbers were significantly higher during the first lockdown than during the second (χ^2^ (1, *n* = 36,245) = 24.774, *p* = 0.00001) and third lockdowns (χ^2^ (1, *n* = 36,547) = 8.7808, *p* = 0.0030). We found a significantly higher number of non-COVID-19 in-hospital deaths vs. discharges during the first lockdown than in the second and third lockdowns (χ^2^ (2, *n* = 26,268) = 7.794, *p* = 0.0203) The number of deaths due to respiratory diseases was significantly higher during the first lockdown than in the second lockdown (χ^2^ (1, *n* = 572) = 8.8185, *p* = 0.0029) and in the third lockdown (χ^2^ (1, *n* = 624) = 9.0381, *p* = 0.0026), and deaths from infectious diseases were higher during the first lockdown than during both the second and third lockdowns (χ^2^ (1, *n* = 566) = 5.9479, *p* = 0.0147, and χ^2^ (1, *n* = 624) = 9.5978, *p* = 0.0019), respectively. The onset of CVA and CVD are abrupt, while respiratory and infectious diseases may have an insidious pattern; this may have led patients to postpone referrals to hospitals to the “last minute” during the first lockdown, perhaps due to fears of contracting COVID-19, and as a result of vague instructions. Citizens and policymakers must be made aware of this point during future pandemics.

## 1. Background

The first case of COVID-19 in Israel was detected in late February 2020 [1]. On 17 March 2020, the Israel Ministry of Health (MOH) made Israel the first country that declared a full emergency lockdown. In a comparison study, the progression of the pandemic was found to be slower in Israel than in Sweden, Greece, and Italy [2]. The MOH instructed citizens to maintain isolation, and moreover, restricted travel. Furthermore, police and inspectors enforced the restrictions during the Passover festival that took place on April 8, and imposed penalties for isolation violators. The lockdown was relaxed, gradually, from 18 April onward. During this first lockdown, citizens were instructed not to visit community clinics unless it was urgent. However, ministerial leaders did not categorically declare hospitals safe for ordinary patients, nor did they disclose that COVID-19 patients bypassed emergency departments and were admitted to specific departments.

In September 2020, during the Jewish festival period, the MOH once again declared a full lockdown that began on September 18, and lasted 21 days. On 27 December 2020, during the Jewish and Christian festivals, the MOH declared a third lockdown that lasted until mid-January. The third lockdown was partial, and included prohibition of gatherings; restaurants and places of entertainment were also closed. In March–April 2020, during the first full lockdown, Magen David Adom (MDA), the Israeli national ambulance service, revealed in reports to the MOH that there was a 22% increase in at-home deaths over the same period in 2019 (1115 vs. 909) [3]. In March 2020, Mafham et al. also assumed that there was a larger number of out-of-hospital deaths due to myocardial complications, after they had witnessed a reduction in in-hospital admissions for myocardial infarction in Britain [4]. Lange et al. showed that, from March 15 to 23 May 2020, ED visits declined by 23% for myocardial infarctions, by 20% for strokes, and by 10% for hyperglycemic crises, compared with the preceding 10-week period (January 5–14 March 2020) [5]. Solomon et al. reported similar findings for myocardial infarctions [6].

These findings have led us to assume that patients who visited hospital EDs during the first COVID-19 lockdown (March–April 2020) may have been those with more urgent cases. From the foregoing, it is possible that patients who were admitted to the hospital during this period may have been generally more ill, and may have chosen to come to the ED at the “last minute”, as Lange et al. assumed [5]. If this assumption is true, it would be interesting to examine if the instructions by the MOH during the first full lockdown for citizens not to visit community clinics on the basis of contagiousness, may have influenced the pattern of visits to EDs, the rates of admission from EDs to hospital wards, and the number of non-COVID-19 in-hospital deaths. One may expect that despite the decline in the number of ED visits, all of these parameters would have risen in comparison to the same period in the last three years, and to the second and third lockdowns, after the MOH encouraged referrals to EDs on the basis of them being declared safe and not contagious.

The Rambam Health Care Campus (RHCC) is a one-thousand-bed tertiary hospital in the north of Israel, with more than 120,000 emergency department (ED) visits each year during 2017–2019. During the first COVID-19 outbreak (March–April 2020), there was an average decline in the number of visits to the ED by 30.2%, and in hospital occupancy by 29.2%, in comparison with the same period in 2019 [7]. Furthermore, according to a report to RHCC management by the oncology division, during the first lockdown, about 9.5% of oncologic patients wanted to reschedule their chemotherapy despite the urgency of their conditions. The oncology department succeeded in reducing this number to 5.1% by reassuring patients through phone calls of the safety of the chemotherapy day care.

The main objective of this study was to examine if the number of non-COVID-19 in-hospital deaths vs. discharges during the first lockdown was higher than those during the same periods in the last three years, and during the second and third lockdowns.

## 2. Materials and Methods

### 2.1. Materials

This was an epidemiological retrospective study that was performed at RHCC. During the first lockdown, positive COVID-19 patients from community clinics bypassed the ED to a “Corona Department”. All patients who were referred to hospital wards from the ED as a result of other diseases were tested for COVID-19 before admission. Any patient who tested positive for COVID-19 was sent instead to the Corona Department. These patients, including those who died from COVID-19, were excluded from the study in order to precisely calculate the number of in-hospital non-COVID-19 deaths vs. discharges. In May 2020, the restrictions were relaxed and, because of a drop in hospital occupancy countrywide, patients were encouraged to visit EDs through a campaign by the MOH. It was emphasized that EDs in Israel were safe, with no danger of contamination. Therefore, it was interesting to compare the pattern of visits and rates of admission and discharges from our ED in March–April 2020, to those in May–June 2020, after the new instructions given by the MOH. We also collected data about the number of non-COVID-19 in-hospital deaths in September–October 2020 (the second lockdown), and in December 2020–January 2021 (the third lockdown). In addition, we drew upon data about the number of non-COVID-19 in-hospital deaths in parallel periods in the three years that preceded the pandemic (2017, 2018, and 2019). We collected data anonymously about the age of every patient who died in each lockdown, in order to increase the accuracy of the results/ conclusions, and to control the variables.

During the first and second lockdowns in RHCC, we used the Allplex™ 2019-nCoV RT-PCR, which is shown to yield the most sensitive results among various RT-PCR kits that were available at the beginning of the COVID-19 pandemic outbreak [8]. In the third lockdown, we used a new version of the same kit, which tests three genes, compared with two genes in the first kit, and is about 3% more sensitive than the first kit.

### 2.2. Methods

All patients’ medical records in RHCC, including the ED, were fully computerized. The business intelligence (BI) computerized software drew on more than fifty parameters from each medical record. It could supply parameters about the profile of each department/unit, measure a patient’s length of stay, time from admission to examination by a physician or a nurse, the number of sophisticated imaging tests ordered, where the patient was discharged to, including death (gate of exit), main diagnosis on admission and on discharge, and secondary diagnoses/operations. The BI software could draw demographic data and allow control of 20 quality parameters that the MOH follows (e.g., time from admission to ED with acute ST elevation MI to cardiac catheterization, etc.). The MD-Clone tool of the BI software supplied higher-resolution information about each patient for further research needs. By supplying data about the cause of a patient’s death as recorded by physicians, the BI software provided us opportunities to include or exclude patients with certain diagnoses, such as COVID-19, and establish cutoffs of certain ages (older than 18) that were relevant to our study. The BI software included an “Operative-Cube Excel” software that could draw data to an Excel worksheet within the BI software, make calculations, compare departments’ activities, distinguish among periods, and build graphs if necessary. From this large database, the data required for this study were harvested with very high resolution and accuracy.

A 2 × 4 chi-square table was used to compare the number of admissions from the ED and the number of discharges in March–April 2020 (the first lockdown), with the corresponding statistics for the same periods in 2019, 2018, and 2017. A 2 × 4 chi-square test was also used to compare the number of non-COVID-19 in-hospital deaths, as well as the number of non-COVID-19 discharges, during the first lockdown with those in the corresponding periods in 2019, 2018, and 2017.

The instruction to citizens during the first lockdown not to visit community clinics due to danger of contagion may have led to fewer visits to the RHCC ED at that time, for fear of developing COVID-19, as mentioned in the Introduction. Thereafter, patients were encouraged to visit EDs since they were declared safe. Thus, we wished to compare the pattern of visits to the ED and non-COVID-19 in-hospital deaths during the first lockdown, to those in the second and third lockdowns, using a 2 × 3 chi-square test.

From the BI software, we drew all causes (main diagnoses) of death for each patient, according to the ICD-11. As mentioned above, all patients with COVID-19 were excluded from the search software. The software output provided resolution at the individual patient level. However, patients with the same diagnosis came in clusters, one following the other without name or ID, and were sent to the Excel worksheet as such. This allowed us to count how many patients died with the same diagnosis. Furthermore, in any category, causes of death were specified. Examples of such diagnoses were bacterial pneumonia cases that were categorized as respiratory diseases, and pulmonary edema cases that were categorized as cardiac disease. Therefore, it was necessary to review each patient and categorize each cause of death in accordance with the underlying disease. Through Operative-Cube Excel that drew data from the BI software, we could count how many patients died from every major diagnosis (see results below), make comparisons, and produce a summarizing graph. We examined the five main causes (diagnoses) of death among the non-COVID-19 patients during the first lockdown, and compared them with the diagnoses during the second and third lockdowns using the 2 × 3 chi-square test.

In order to minimize the variables and render the results more accurate, we used MD-Clone of the BI software to determine exact age in day-to-day resolution. In order to compare the average ages of those who died in each lockdown, a one-way ANOVA test was performed.

No data from the business intelligence statistical software revealed patient details. We were granted an exemption by the Rambam Health Care Campus Helsinki Committee (IRB).

## 3. Results

Comparing the admissions and discharges from the ED in March–April 2020 (first lockdown) with the same variables in the same periods in 2019, 2018, and 2017, we found a significantly higher rate of admissions from the ED in March–April 2020 (χ^2^ (3, *n* = 84,972) = 77.18, *p* < 0.00001). A higher admission rate was also observed in the second and the third lockdowns than in the corresponding periods of the three previous years (Table 1).

Separately, we compared the number of admissions and discharges from the ED during the first lockdown period with the same period in each of the three previous years. The results showed a significantly higher rate of admissions vs. discharges from the ED during the first lockdown (32.9%) than in the same period in 2019 (29.7%; χ^2^ (3, *n* = 39,065) = 45.665, *p* < 0.00001), 2018 (29.4%; χ^2^ (3, *n* = 38,984) = 56.7568, *p* < 0.00001), and 2017 (29.2%; χ^2^ (3, *n* = 38,600) = 61.2805, *p* < 0.00001). We found no significant differences in the number of admissions vs. discharges from the ED in the same periods among the years 2017, 2018, and 2019 (χ^2^ (2, *n* = 69,158) = 1.5628, *p* = 0.4577).

There was a significantly higher admission rate during the first lockdown than in both the second (χ^2^ (1, *n* = 36,245) = 24.774, *p* = 0.00001) and third lockdowns (χ^2^ (1, *n* = 36,547) = 8.7808, *p* = 0.0030). There was also a significantly higher admission rate during the third lockdown than the second lockdown (χ^2^ (1, *n* = 41,124) = 4.7033, *p* = 0.0310). It would have been interesting if the corresponding three periods in the three previous years behaved the same way. However, comparing the three periods within each year separately, we found no similar pattern of behavior (see Table 1).

Comparing the number of non-COVID-19 in-hospital deaths to the number of non-COVID-19 discharges from hospital wards during the first lockdown to those in the same periods in 2019, 2018, and 2017, there was a significantly higher number of non-COVID-19 deaths vs. discharges during the first lockdown (March–April 2020) (χ^2^ (3, *n* = 37,570) = 20.9492, *p* < 0.0001). In order to confirm this result, we compared the non-COVID-19 in-hospital deaths vs. discharges during the first lockdown with those in the same period for each year separately; again, the results revealed a significantly higher number of in-hospital deaths vs. discharges during the first lockdown (*p* < 0.0001) (Table 2).

On 18 September 2020, due to the upcoming Jewish festivals and in light of the gradual spread of COVID-19, a full second lockdown was imposed for 21 days in order to prevent the spread of COVID-19 among extended family members. We compared the number of non-COVID-19 deaths vs. non-COVID-19 discharges during the second lockdown with the number of deaths vs. discharges during the corresponding periods in the previous three years (2017–19). The results revealed a non-significant difference among the four periods (Table 2).

A third lockdown, which was a partial one, was imposed in December 2020–January 2021 during the Jewish and Christian festivals. Comparing the number of deaths and discharges among non-COVID-19 patients in the third lockdown with those in the corresponding periods in the previous three years (2017, 2018, and 2019), we again found a non-significant difference in the number of in-hospital deaths vs. discharges among the four periods (Table 2).

In May–June 2020 (as well as in July–August 2020), after the first lockdown, the restrictions were relaxed. As a result of a drastic reduction in the number of visits to EDs during the first lockdown, ill citizens were encouraged to go to hospitals through emphasizing the safety of hospitals’ EDs and wards, as mentioned above. Comparing the number of non-COVID-19 deaths and discharges in March–April 2020 (the first lockdown) to those in May–June 2020, as well as in Jul–Aug 2020, on a bi-monthly basis, we found a significantly higher non-COVID-19 in-hospital death number vs. discharges in March–April 2020 (the first lockdown) compared with the two periods that followed (after relaxation of the lockdown, and when there were clear government instructions) (χ^2^ (2, *n* = 26,734) = 25.3143, *p* < 0.00001) (Table 3). In order to further confirm the results, we separately compared the number of non-COVID-19 in-hospital deaths vs. discharges in March–April 2020 (300 deaths vs. 7300 discharges) to those in May–June 2020 (249 deaths vs. 9153 discharges) and July–August 2020 (285 deaths vs. 9447 discharges). The results also showed a significantly higher number of non-COVID-19 deaths in March–April 2022 compared to May–June 2020 and July-August 2020 (χ^2^ (1, *n* = 17,002) = 22.6951, *p* < 0.00001, and χ^2^ (1, *n* = 17,332) = 13.5838, *p* = 0.00002, respectively) (Table 3).

We found a significantly higher number of non-COVID-19 in-hospital deaths vs. discharges during the first lockdown compared to the second and third lockdowns (χ^2^ (2, *n* = 26,268) = 7.794, *p* = 0.0203) (Table 3). The non-COVID-19 deaths vs. discharges during the first lockdown were also significantly higher than the non-COVID-19 deaths vs. discharges during the second lockdown (χ^2^ (1, *n* = 16,259) = 7.749, *p* = 0.005). However, there was no difference in non-COVID-19 deaths vs. discharges between the second and third lockdowns—χ^2^ (1, *n* = 17,668) = 2.086, *p* = 0.0938 (Table 3).

In order to increase the accuracy of the study and the conclusions, we checked for differences in age groups among the non-COVID-19 deaths in the three lockdowns by means of a one-way ANOVA test. The results revealed a non-significant difference among the age groups in the three lockdowns (F(2, 893) = [F-2.6093], *p* = 0.0741).

Of the 261 causes of death according to the ICD-11, the five leading reasons for death among non-COVID-19 patients were the following: respiratory disease/complications (e.g., COPD, severe bacterial pneumonia, asthma, COPD exacerbation, viral non-COVID-19 “atypical” pneumonia, empyema, lung abscess. resp. system/other chest symptoms, and spontaneous pneumothorax), cardiovascular disease, (CVD)/complications (e.g., acute myocardial infarction, ventricular fibrillation, pulmonary edema, cardiogenic shock, cardiac dysrhythmia, aortic aneurysm, cardiomyopathy, congestive heart failure, and myocarditis), cerebrovascular disease (e.g., spontaneous hemorrhagic CVA, ischemic CVA, and encephalomyelitis), neoplasia/complications (such as metastatic neoplasia, end-stage neoplasia, neoplasia of any organ, hematologic neoplasia, aplastic anemia, and lymphomas), and infectious diseases (e.g., septic shock, neutropenic sepsis, meningitis, other tissues infections, sepsis, urinary tract infection, urosepsis, and subacute/acute endocarditis).

**Figure 1:**
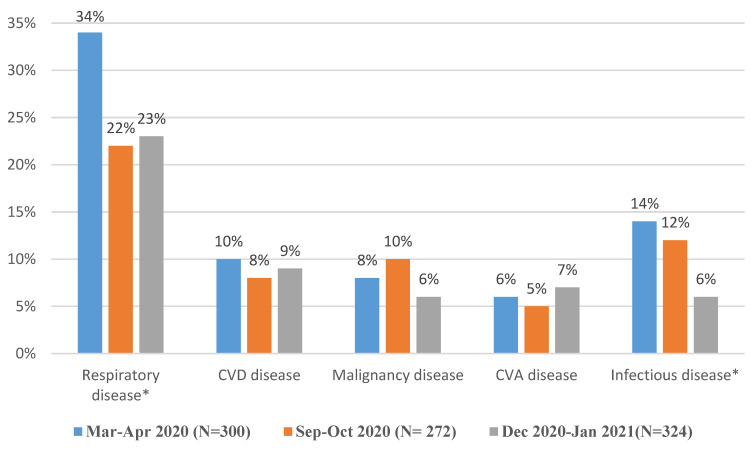
The prevalence (percent) of main five causes of death among non-COVID-19 patients during the first, second, and third lockdowns. * Indicates statistically significant difference among the three periods; *n* = the total number of non-COVID-19 deaths.

We found no significant differences in the number of deaths due to CVD, malignancy, and CVA, among the three lockdowns. We obtained the following results: for CVD (χ^2^ (2, *n* = 896) = 0.8289, *p* = 0.6606), for malignancy (χ^2^ (2, *n* = 896) = 3.4479, *p* = 0.1783), and for CVA (χ^2^ (2, + 896) = 0.8741, *p* = 0.6459). Separately, there were also non-significant differences between the first lockdown and the second and third lockdowns for each disease category (Figure 1).

Comparing the differences in the causes of death among the three lockdowns, we found a significantly higher number of deaths due to respiratory diseases/complications during the first lockdown than in the second lockdown (χ^2^ (1, *n* = 572) = 8.8185, *p* = 0.0029) and third lockdown (χ^2^ (1, *n* = 624) = 9.0381, *p* = 0.0026). However, there were no significant differences in the number of deaths due to respiratory diseases/complications between the second and third lockdowns (χ^2^ (1, *n* = 596) = 0.0005, *p* = 0.9826).

In addition, comparing the causes of death among the three lockdowns revealed a significantly higher number of deaths due to infectious diseases/complications during the first lockdown than during both the second and third lockdowns (χ^2^ (1, *n* = 566) = 5.9479, *p* = 0.0147, and χ^2^ (1, *n* = 624) = 9.5978, *p* = 0.0019, respectively). However, there was a non-significant difference in the prevalence of infectious diseases/complications between the second and third lockdowns (χ^2^ (1, *n* = 590) = 0.1091, *p* = 0.7411) (Figure 1).

## 4. Discussion

There was a significant decrease in the number of visits to our ED in March–April 2020 (first lockdown), compared with the same period in the previous three years. There was a significant decrease in the number of visits to our ED in March–April 2020 (first lockdown), compared with the second and third lockdowns. There was a significant increase in the percentage of hospitalized and discharged patients from our ED in the first lockdown, compared with the second and third lockdowns, as well as with the four months after the first lockdown.

A previous study at RHCC showed a significant decrease in the number of visits to the ED, but an increase in the percentage of ED deaths in the first lockdown compared with the same period a year before [7]. The authors assumed that the patients who applied for admission to the ED in March–April 2020 had probably more complex conditions and may, as other authors suggested, have gone to the hospital too late [5,9].

Although there was a higher number of referrals to the ED in both the second lockdown (23,222) and the third lockdown (22,766) compared with the first lockdown (15,834), we found a higher significant rate of admissions (admissions vs. discharges) from the ED in the third lockdown (*p* = 0.031) relative to the second lockdown. Perhaps, the significantly higher rate of admission during the third lockdown (December–January) may be explained by winter diseases, or the low occupancy of our hospital wards that winter (78.2%), which led to higher admissions of less severe cases from the ED; such cases, before the pandemic when the occupancy of the hospital wards sometimes exceeded 100%, ordinarily would have been discharged for further investigation at community clinics. We did not witness the same pattern when we compared the third lockdown with each of the same three periods in the years 2017, 2018, and 2019 separately.

We also found a significant increase in the number of non-COVID-19 deaths vs. discharges in our hospital during the first lockdown (March–April 2020) compared to the same periods in the previous three years. There was a significant increase in the number of non-COVID-19 in-hospital deaths in March–April 2020 on a bi-monthly basis, compared to May–August 2020, when patients were encouraged to visit EDs. There was a significant increase in the non-COVID-19 in-hospital deaths during the first lockdown period compared to the second and third lockdowns, together and separately.

In the early stages of the pandemic, some reports from the USA indicated a higher number of in-hospital deaths vs. discharges between March and April 2020; however, they could not estimate if some of these patients were COVID-19-positive or not [10,11]. In our study, because all hospitalized patients were tested for COVID-19, and COVID-19 patients bypassed our ED, we were more certain about the cause of death among hospitalized patients. In addition, other researchers showed an increase in mortality from heart disease, diabetes, and other diseases. However, they could not determine the extent to which these trends represented non-respiratory manifestations of COVID-19, or were secondary to mortality that was caused by disruptions in society that diminished or delayed access to health care [12].

Catalano et al., analyzing local data, reported an increase in the number of non-COVID-19 deaths among the general population in Norway during the early weeks of the pandemic [13]. However, these results relied on general undetailed data that were published in the country of study. By obtaining data from the CDC during the first three months of the pandemic, Jacobson showed similar results [14]. Another study conducted in Portugal also showed significantly high mortality during the first month of the pandemic, mostly among people aged over 75. Only 50% of mortality was registered as being directly due to COVID-19 [15]. Other researchers in the UK found a higher mortality rate during the COVID-19 pandemic, but they also suggested that further investigation should be undertaken in order to ascertain how many of the patients may have died from COVID-19 [16]. All of these findings may have led to the assumption that people were referred to EDs “too late” or “at the last minute” [5]. We found no studies on PubMed or Google Scholar that specifically examined if the non-COVID-19 in-hospital death rate at the onset of the pandemic was higher than those in previous years.

Compared to previous studies, we went a step further to specifically measure if there were higher non-COVID-19 in-hospital deaths during the first months of the pandemic, compared to the same periods in the previous three years, and to the subsequent lockdowns. Our findings may shed some light on many people’s “gut feeling” concerning the higher rate of non-COVID-19 deaths.

During the first months of the pandemic, citizens and governments were anxious about the sequences of the virus. Fear led people to not want to visit EDs and hospitals in many countries around the world, as was the case in Israel and, specifically, our hospital. Moreover, when the Israeli MOH instructed citizens to visit community clinics only for urgent cases, they were unaware that citizens could perceive hospitals as a dangerous place to visit. This could explain the drastic decrease in the number of visits to EDs during the COVID-19 epidemic, compared to the increase in the number of visits to EDs that we experienced during the H1N1 pandemic of 2008–9 [7].

Some studies reported a decrease in the number of in-hospital cardiac and cerebral events during the pandemic [17,18]. In our study, we examined the behavior of patients and the MOH for 10 months and over three lockdowns. Based on the above-mentioned results and other results published by other researchers about the rates of death from CVD and CVA, we examined and compared the five main causes of death among non-COVID-19 patients during the three lockdowns. We found no significant differences in the rates of death due to CVD, malignancies, and CVA, between the three lockdowns. We assumed that this stemmed from the urgency and dramatic presentations of these diseases (cardiac events, CVA, and end-stage malignancies). On the other hand, other diseases, such as respiratory and infectious diseases, may gradually aggravate, and patients may postpone going to the hospital until the last minute. This may explain the higher number of deaths due to respiratory and infectious complications during the first lockdown compared to the second and third lockdowns. It may also explain the initial findings about a higher number of total deaths vs. discharges during the first lockdown than in the same periods in the previous three years, and in the two subsequent lockdowns.

After the first lockdown, there was a decrease in the number of visits to our ED, as occurred in many other countries [19,20]. The results showed also that after the first lockdown, there was a gradual but rapid increase in the number of admissions to the hospital from May until December 2020 (including the second and third lockdowns). Accordingly, hospital occupancy increased. This phenomenon may be explained by two situations. Firstly, it could be that after the MOH campaign that encouraged citizens to go to EDs if the need arises, as in regular days, there was a gradual increase in the number of referrals to our ED and therefore, an increase in hospital occupancy. Secondly, at the onset of a crisis, such as the COVID-19 pandemic, most people use mental and physical resources for short-term survival in very stressful situations. During the initial stages of a crisis, misinformation may be rampant, and there may be a lack of consistent guidance from leadership about what effective actions to take. However, as the crisis persists, people adopt a different coping style, which may lead to “pandemic fatigue”, and low motivation may prevail [21]. This could partly explain the population’s about-face to visit community clinics and honor referrals to EDs in the subsequent stages of the pandemic. Yet, the number of referrals to our ED during the second half of 2020 remained significantly lower than that during the same period in the previous three years, a finding that may explain the higher rate of admissions from the ED during the third lockdown in comparison to the second lockdown.

## 5. Conclusions

With globalization, there may be future pandemic outbreaks. Pandemics may put governments and ministries of health in a state of fear, as may have been the case in Israel and in many other countries. This may lead to a failure to consider many important factors, some of which may be related to the behavior of the public during visits to Eds, and may adversely affect their health. This failure, together with misinformation disseminated via social media, may add to people’s fear [22]. We suggest that policymakers consider the possible implications of their policies and provide clear instructions to the masses, as well as address fake news that circulates via social media [23].

There may be some limitations to this study. Although our hospital is the largest in northern Israel, with thousands of visits to its ED every month, it can be argued that RHCC does not necessarily represent what happens at the same time in other hospitals in Israel and elsewhere. Given what is available in the literature, and although we think the situation in Israel may have been similar to the situations in other countries, we encourage other hospitals to evaluate the mortality rates among non-COVID-19 patients in the first months of the pandemic, in order to confirm or disprove our hypothesis.

Another limitation of the study is the precise cause or diagnosis among the five main causes of death. There are some patients who died with a diagnosis of “dysrhythmia” or “other respiratory symptom”, but with no mention of the exact kind of dysthymia or the specific respiratory disease. This may have happened because the ICD-11 does not always offer an exact diagnosis, and physicians tend to choose the closest diagnosis that reflects the reasons for death. Although we may still use the system to categorize causes of death without influencing the results, we should consider this point in further studies.

## Figures and Tables

**Table 1:** Number of admissions vs. discharges from the emergency department.

	COVID-19 Period	Non-COVID-19 Period			
	**Mar.–Apr. 2020** **First Lockdown**	**Mar.–Apr. 2019**	**Mar.–Apr. 2018**	**Mar.–Apr. 2017**	
Admissions from ED	5217	6903	6797	6650	χ^2^(3, *n* = 84,972) = 77.18 *p* < 0.00001
Discharges from ED	10,617	16,319	16,353	16,116	
% Admissions from total visits	32.9% ^a I^	29.7% ^b I^	29.4% ^b II^	29.2% ^b I^	
	**Sept.–Oct. 2020** **Second lockdown**	**Sept.–Oct. 2019**	**Sept.–Oct. 2018**	**Sept.–Oct. 2017**	
Admissions from ED	6225	6868	6978	6855	χ^2^(3, *n* = 94,458) = 63.85 *p* < 0.00001
Discharges from ED	14,186	18,271	17,277	17,798	
% Admissions from total visits	30.5% ^a III^	27.3% ^b II^	27.6% ^b II^	27.8% ^b II^	
	**Dec. 2020–Jan. 2021** **Third lockdown**	**Dec. 2019–Jan. 2020**	**Dec. 2018–Jan. 2019**	**Dec. 2017–Jan. 2018**	
Admissions from ED	6522	7020	7041	7236	χ^2^(3, *n* = 90,985) = 17.62 *p* = 0.0005
Discharges from ED	14,191	16,143	15,752	17,080	
% Admissions from total visits	31.5 ^a II^	30.3% ^bc I^	30.9% ^ac I^	29.8% ^bd I^	
Total P	χ^2^(2, *n* = 56,958) = 24.82 *p* < 0.00001	χ^2^(2, *n* = 71,524) = 59.37 *p* < 0.00001	χ^2^(2, *n* = 70,198) = 26.78 *p* < 0.00001	χ^2^(2, *n* = 71,735) = 24.13 *p* < 0.00001	

Note. Different letters (a,b,c,d) mark significant differences across rows for each of the three periods separately (Mar.–Apr., Sept.–Oct., Dec.–Jan). Different Roman numerals (I, II, III) mark significant differences across columns for each of the four years separately (2020, 2019, 2018, 2017).

**Table 2:** In-hospital non-COVID-19 deaths vs. non-COVID-19 discharges from hospital departments.

	**Mar.–Apr. 2020** **First Lockdown**	**Mar.–Apr. 2019**	**Mar.–Apr. 2018**	**Mar.–Apr. 2017**	
Non COVID-19 in-hospital deaths	300 (4.10%)	350 (3.59%)	298 (3.06%)	280 (2.92%)	*p* = 0.0001
Non COVID-19 discharges from hospital	7300	9731	9723	9588	
	**Sept.–Oct. 2020 second lockdown**	**Sept.–Oct. 2019**	**Sept.–Oct. 2018**	**Sept.–Oct. 2017**	
Non COVID-19 in-hospital deaths	272 (3.24%)	270 (2.81%)	287 (3.01)	276(2.91%)	*p* = 0.4147
Non COVID-19 discharges from hospital	8387	9577	9525	9478	
	**Dec. 2020–Jan. 2021 Third lockdown**	**Dec. 2019–Jan. 2020**	**Dec. 2018–Jan. 2019**	**Dec. 2017–Jan. 2018**	
Non COVID-19 in-hospital deaths	324 (3.73%)	329 (3.20%)	337 (3.24%)	376 (3.77%)	*p* = 0.3686
Non COVID-19 discharges from hospital	8685	10262	10379	9956	

**Table 3:** Non-COVID-19 in-hospital deaths during the first lockdown compared to May–June and July–August 2020, and the first compared to the second and third lockdowns.

	**Mar.–Apr. 2020 First Lockdown**	**May–Jun. 2020**	**Jul.–Aug. 2020**	
Non-COVID-19 in-hospital deaths	300 (4.10%)	249 (2.72%)	285 (3.01%)	*p* < 0.00001
Non-COVID-19 discharges from hospital	7300	9153	9447	
	**Mar.–Apr. 2020** **First lockdown**	**Sept.–Oct. 2020** **Second lockdown**	**Dec. 2020–Jan. 2021** **Third lockdown**	
Non-COVID-19 in-hospital deaths	300 (4.10%)	272 (3.24%)	324 (3.73%)	*p* = 0.0203
Non-COVID-19 discharges from hospital	7300	8387	8685	

## Data Availability

The data presented in this study are available on request from the corresponding author.
